# P-1572. Critical care billing and G0545 code impact on Infectious Diseases revenue

**DOI:** 10.1093/ofid/ofaf695.1752

**Published:** 2026-01-11

**Authors:** Gina Maki, Cassandra Salgado, Laura Sublett, Marcus Zervos, John E McKinnon

**Affiliations:** Henry Ford Health System, Detroit, MI; Medical University of South Carolina, Charleston, SC; Medical University of South Carolina, Charleston, SC; Henry Ford Hospital, Detroit, Michigan; Division of Infectious Diseases, Department of Medicine, Medical University of South Carolina,, Charleston, SC

## Abstract

**Background:**

Infectious Diseases (ID) divisions aim to optimize documentation and proper reimbursement for inpatient services with complex patients. Utilization of critical care billing and new add-on G0545 codes have provided new revenue sources but require improved and specific documentation for their use.

**Methods:**

We instituted a documentation, billing, and coding program at two academic centers: the ID division at Henry Ford Health (HFH) (2017-present) and at the Medical University of South Carolina (MUSC) (2023-present). These programs were enacted by ID providers directing education, billing, documentation, and coding services with a particular focus on critical care billing and the adoption of new coding rules such as G0545 add-on code. Revenue value units (RVUs) are considered reimbursed at 2025 CMS rate.

**Results:**

MUSC ID providers adopted using critical care billing in 2023 with an increased code use from 0 in 2022 to 53 in 2023, 91 in 2024 and 87 for 2025 thus far. This represents RVUs and CMS dollar totals of 409.5 and $13,231.15 in 2024, and 391 and $12,648 in 2025. The adoption of G0545 add on code thus far has been utilized on 1132 notes, a coding rate of 13.15%, and represents an additional 1007 RVUs and $32,576 in the first 3 months of use. At HFH, critical care billing was initiated in April 2020 with increased utilization for critical care notes up to over 330 notes by October 2020 during the COVID-19 pandemic. Since 2022, level 1 and 2 critical care billing has average 610 and 55 notes per year, representing a yearly average in RVU production of 3115 and $100,786 per year by 2025 CMS rates. G0545 coding has been applied on 1582 notes, at a rate of 55%, generating 1407.8 RVUs and $45,548 for the first quarter of 2025.

**Conclusion:**

Improved training, documentation and proper billing for ID services allows adoption and use of critical care billing codes and new G0545 add-on codes leading to increases in the value generated per service performed by ID physicians. Further expansion of the properly directed use of these codes can have significant positive effects on ID divisional revenue and reimbursement.

**Disclosures:**

Marcus Zervos, MD, merck: Honoraria John E. McKinnon, MD, MSc, FIDSA, EMD Serono: Advisor/Consultant

